# Identification of a functional peptide of a probiotic bacterium-derived protein for the sustained effect on preventing colitis

**DOI:** 10.1080/19490976.2023.2264456

**Published:** 2023-10-10

**Authors:** Harpreet Kaur, Syed Azmal Ali, Sarah P. Short, Christopher S. Williams, Jeremy A. Goettel, M. Kay Washington, Richard M. Peek, Sari A. Acra, Fang Yan

**Affiliations:** a Department of Pediatrics, Vanderbilt University Medical Center, Nashville, TN, USA; b Division of Proteomics of Stem Cell and Cancer, German Cancer Research Center, Heidelberg, Germany; c Department of Medicine, Vanderbilt University Medical Center, Nashville, TN, USA; d Department of Pathology, Microbiology and Immunology, Vanderbilt University Medical Center, Nashville, TN, USA; e Center for Mucosal Inflammation and Cancer, Vanderbilt University Medical Center, Nashville, TN, USA; f Vanderbilt Institute for Infection, Immunology and Inflammation, Vanderbilt University Medical Center, Nashville, TN, USA; g Department of Cell and Developmental Biology, Vanderbilt University, Nashville, TN, USA

**Keywords:** Histone methyltransferase, intestinal epithelial cell, p40, Setd1β, TGFβ

## Abstract

Several probiotic-derived factors have been identified as effectors of probiotics for exerting beneficial effects on the host. However, there is a paucity of studies to elucidate mechanisms of their functions. p40, a secretory protein, is originally isolated from a probiotic bacterium, *Lactobacillus rhamnosus* GG. Thus, this study aimed to apply structure-functional analysis to define the functional peptide of p40 that modulates the epigenetic program in intestinal epithelial cells for sustained prevention of colitis. *In silico* analysis revealed that p40 is composed of a signal peptide (1–28 residues) followed by a coiled-coil domain with uncharacterized function on the N-terminus, a linker region, and a β-sheet domain with high homology to CHAP on the C-terminus. Based on the p40 three-dimensional structure model, two recombinant p40 peptides were generated, p40N120 (28–120 residues) and p40N180 (28–180 residues) that contain first two and first three coiled coils, respectively. Compared to full-length p40 (p40F) and p40N180, p40N120 showed similar or higher effects on up-regulating expression of *Setd1b* (encoding a methyltransferase), promoting mono- and trimethylation of histone 3 on lysine 4 (H3K4me1/3), and enhancing *Tgfb* gene expression and protein production that leads to SMAD2 phosphorylation in human colonoids and a mouse colonic epithelial cell line. Furthermore, supplementation with p40F and p40N120 in early life increased H3K4me1, *Tgfb* expression and differentiation of regulatory T cells (Tregs) in the colon, and mitigated disruption of epithelial barrier and inflammation induced by DSS in adult mice. This study reveals the structural feature of p40 and identifies a functional peptide of p40 that could maintain intestinal homeostasis.

## Introduction

The mutualistic relationship between the gut microbiota and host is established by the contribution of the host to providing a nutrient-rich environment for microbial growth and function as well as the beneficial effects of microbiota on maintaining host health.^1,2^ Dysregulation of the microbial-host interactions is a risk factor for, or is a consequence of several diseases, including inflammatory bowel disease (IBD)^3,4^ and metabolic disorders.^5,6^ Therefore, microbiota-targeting therapies have emerged as potential strategies for disease prevention and treatment. Probiotics, which are microorganisms beneficial to host health, have shown promising beneficial effects in human, animal and *in vitro* studies.^7,8^ However, the efficacy of probiotics in clinical applications is poorly understood.^9,10^ In addition to wide variations in probiotic strain selection and dosing in probiotic clinical trials, uncertain clinical outcomes result from the lack of precision in host variables, including their overall health status, gut microbiome profile, and diet,^7^ which may limit probiotic bioavailability and biopharmacology in the gastrointestinal tract. To address these concerns, recent research has demonstrated a molecular mechanism underlying the effects of probiotics: production of functional factors, which include secretory products and metabolites, and bacterial fractions.^8,11^ Various functions of these factors have been identified, such as protection of intestinal mucosal barrier, stimulation of anti-inflammatory responses and inhibition of pro-inflammatory responses.^8,11^ Notably, the use of these functional factors as therapeutic targets has the potential to circumvent the clinical restrictions of direct probiotic usage.

p40, a secretory protein, was originally isolated from culture supernatants of a commonly used probiotic bacterium, *Lactobacillus rhamnosus* GG (LGG).^12^ Genes encoding homologues to p40 are mainly present in *L. casei/paracasei/rhamnosus* phylogenomic groups.^13^ We have demonstrated several biological functions of p40, such as transactivation of epidermal growth factor (EGF) receptor in intestinal epithelial cells that mediates the immediate effects on preserving the intestinal barrier and stimulating protective immunity for ameliorating experimental colitis in mice.^14–17^ Our recent work demonstrated long-lasting effects of p40 on prevention of colitis^18^ and revealed a significant mechanism of p40 for this effect: p40 supplementation in early life induces sustained increase in TGFβ production in intestinal epithelial cells in adult mice, leading to Treg cell induction and protection against colitis in adult mice. The sustained effects of p40 on TGFβ production is mediated by stimulating the expression of a methyltransferase, Setd1β, in intestinal epithelial cells.^19^ These findings provide mechanistic insight for understanding of life-long health outcomes in humans and animals conferred by colonization of the gut microbiota during a critical window of early life.^20^

Currently, how bacterium-derived proteins such as p40 regulate biological processes in host cells are not fully understood. One potential approach is to elucidate the structure-functional relationship of these proteins by using a sequence-based structural reconstruction model. In addition, the unique characteristics of short peptides, such as those with high efficiency of crossing cell membranes and reaching intracellular targets, give promise for innovative biotherapies. Here, we applied comparative genomic analysis and structure-functional assays to identify the functional peptide of p40 for epigenetic modification of intestinal epithelial cells for the long-lasting effects on prevention of colitis. By using the accurate protein structure prediction programs, SWISS-MODEL, Alpha-fold, and i-TASSER, we have found that p40 is composed of a coiled-coil domain in the N-terminus and a β-sheet structure in the C-terminus. p40N120 recombinant peptide (28–120 aa) containing the first two alpha helices showed the same or higher regulatory effects on epigenetic imprint on *Tgfb1* in intestinal epithelial cells, leading to sustained protection against colitis compared to the full-length p40 (p40F) and p40N180 peptide (28–180 aa) with the first three alpha helices. These findings suggest that functional peptides of probiotic-derived factors may serve as next-generation microbiota-based therapies for promoting intestinal health.

## Materials and methods

### Multiple sequence alignment and three-dimensional structure modeling of p40

By using the published p40 amino acid sequence,^12^ multiple sequence alignment (MSA) of p40 and functional protein sequences were verified by the alignment using ClustalW algorithm in Molecular Evolutionary Genetics Analysis version 11 (MEGA 11).^21^ The family of cell wall hydrolase proteins and CHAP domain-containing proteins were collected based on high sequence similarity from NCBI data for MSA. To ensure the reliability and biological significance of our MSA, a stringent set of statistical criteria was employed, including implementing an 80% sequence identity threshold, utilizing an E-value of 1e-5 and a bit score threshold of 50 to highlight sequences with significant alignment, and conservation scores for residue-level analysis (Supplemental data 1). In addition, alignment length, coverage, and the presence of gaps or insertions to ensure alignment quality were assessed. To avoid redundancy, cluster analysis and prioritized sequences with well-defined functional annotations were conducted.

Modeling of p40 three-dimensional structure was performed using three different computational tools, SWISS-MODEL, Alpha-fold, and i-TASSER. First, to assess and predict the structure of p40 protein, we analyzed p40 three-dimensional model using SWISS-MODEL.^22^ This model was derived from homology modeling of PcsB, a 45 kDa secreted protein from *Streptococcus pneumoniae* sequence (PDB: 4cgk.1), used as a template.^23^ Further, p40 protein sequence was then submitted to Alpha-Fold (https://alphafold.ebi.ac.uk/)^24^ to predict structure validation. The predicted structure and function were further curated using different scores generated from i-TASSER (https://zhanggroup.org/I-TASSER/).^25^ The structure selection for i-TASSER was based on C-Score, RMSD-Score and TM-Score. The alpha helix and beta sheet motifs and known interacting regions were visualized using Jmol, an open-source Java viewer for chemical structures in 3D (http://www.jmol.org/).

### Production of recombinant p40 peptides and purification of p40F

p40 N-terminal 1–28 residue serves as a signal sequence for protein secretion. We generated two recombinant p40 peptides: N-terminal 28–120 residues (p40N120) with α1 and α2 and N-terminal 28–180 residues (p40N180) with α1, α2 and α3. Briefly, genes encoding these two peptides were synthesized and cloned into pET30a DNA vector carrying an N-terminal His Tag/thrombin/S Tag/enterokinase configuration (#69909, MilliporeSigma, St. Louis, MO) at Nde I and Hind III restriction sites. The expression plasmids were transformed into *E. coli* BL21 (DE3) competent expression cells (#EC0114, Thermo Fisher Scientific Inc). Recombinant peptides were purified using Ni-IDA column (#PR-HTK, MoBiTec). Peptides bound to the column were eluted by using urea buffer.

*Lactobacillus rhamnosus* GG (LGG) (#53103; American Type Culture Collection, Manassas, VA) was cultured in Lactobacillus MRS broth. According to the published method,^12^ p40F was purified from LGG culture supernatant using UNOsphere S ion ex-change media (#1560113, Bio-Rad Laboratories, Hercules, CA), eluted using NaCl containing Tris buffer, and concentrated using two centrifugal filter devices with molecular weight cutoff of 30 and 50 kDa (EMD Millipore Corporation, Billerica, MA).

Production of p40N120 and p40N180 and p40F isolation were validated by staining of SDS-gel using colloidal blue stain kit (#LC6025, Thermo Fisher Scientific Inc.) and Western blot analysis using an anti-p40 antibody. p40N120 and p40N180 and p40F were saved at −80°C.

### Human colonoid culture and treatment

Adult human normal colonic tissues were obtained through the Vanderbilt Cooperative Human Tissue Network. As described before,^26^ crypts were collected and suspended in growth factor reduced Matrigel (#356230, Corning Incorporated, Corning, NY) and overlaid with media IntestiCult^TM^ Organoid Growth Medium (#06010, STEMCELL Technologies, WA, USA). For colonoid subculture, colonoids were broken down by vigorously pipetting and cultured as above. All colonoids became budding at 3–4 days of culture. Colonoid treatment was performed by supplementing with p40F, p40N180, or p40N120 at 100 ng/ml in medium for 48 h before stopping experiments. Colonoids were removed from culture dishes by using cell recovery solution (354253, Corning), and collected organoids were fixed in 10% neutral buffered formalin for 20 min followed by embedding in 2% agarose. Agarose plugs containing organoids were then embedded in paraffin to allow hematoxylin and eosin staining and immunostaining.

### Cell culture and treatment

Young adult mouse colon (YAMC) epithelial cell line is a conditional immortalized cell line generated from immortomice^27^ harboring thermolabile simian virus 40 (SV40) large tumor antigen (TAg) from a SV40 strain, tsA58.21. Cell proliferation requires the expression of SV40 TAg, which is induced by an IFN-γ-inducible H-2Kb promoter at the permissive temperature (33°C). YAMC cells were maintained in RPMI 1640 medium supplemented with 10% fetal bovine serum (FBS), 5 U/mL of murine IFN-γ, 100 U/mL penicillin and streptomycin at 33°C with 5% CO2. Cells were cultured in RPMI 1640 medium containing 1% FBS and 100 U/mL penicillin and streptomycin as a serum-starve medium for 18 h at 37°C before treatment with p40F, p40N180 and p40N120. RNA and protein samples from cells and culture supernatants were collected.

### Mouse treatment

All animal work was performed with approval from the Vanderbilt University Medical Center Institutional Animal Care and Used committee following Animal Research: Reporting of *In Vivo* Experiments standards. Wild-type C57BL/6J (000664; Jackson Laboratory, Bar Harbor, ME) and Foxp3-GFP transgenic mice on a Balb/c background (006769; Jackson Laboratory) were used for this study. For each experiment, littermate female mice grew in the same cage were mated with one male mouse and were housed until delivery. p40F and p40N120 were encapsulated in pectin solution in water (2.0% w/v) at concentrations of 0.5 or 1 μg/hydrogel and coated with zein solution (1.0% w/v) and CaCl_2_ (0.5% w/v) in 85% ethanol solution, as described in published paper.^18^ As negative controls, pectin/zein hydrogels without any p40 products were prepared. Mice in each litter was supplemented with p40F or p40N120 containing hydrogels or control hydrogels at the following dosages: of 0.5 μg/day (postnatal days 2–6), 1.0 μg/day (days 7–13), 1.5 μg/day (days 14–21).

### Colitis induction and assessment

Colitis was induced in 8-week-old C57BL/6 mice by the administration of 3% DSS (molecular weight 36–50 kDa, MP Biomedicals, CA, USA) in drinking water for 4 days. Mice supplemented with drinking water were used as control. Paraffin-embedded Swiss-rolled colon tissue sections were stained with hematoxylin and eosin for light microscopic examination to assess colon injury and inflammation. Samples from the entire colon were examined by a pathologist blinded to treatment conditions. A modified combined scoring system^28^ including degree of inflammation (scale of 0–3) and crypt damage (0–4), percentage of area involved by inflammation (0–4) and crypt damage (0–4), and depth of inflammation (0–3) was applied for assessing intestinal injury and colitis.

### Flow cytometric analysis of differentiation of tregs

The colonic tissues from Foxp3-GFP mice were prepared for processing single-cell suspensions of lymphocytes from colonic lamina propria, as described previously.^18^ Cells were labeled with LIVE/DEAD fixable viability dyes (#2443413, Invitrogen, Waltham, MA) and PE-Cy5-anti-CD4 (#100410, BioLegend, San Diego, CA) at room temperature. Then, cells were analyzed using multi-color flow cytometry, a BD LSRII system (BD Biosciences) to determine the percentage of GFP (Foxp3 expression) and PE-Cy5.5 (CD4 expression) double positive cells within live cell population. Each sample contained lymphocytes from 2 to 3 mice with the same treatment.

### The enzyme-linked immunosorbent assay (ELISA)

The level of TGFβ in supernatants of cultured YAMC cells was measured using a mouse TGF-β ELISA kit (#88-8350-88, Invitrogen), according to the manufacturer’s instruction. Purified TGFβ was used to generate the standard concentration curve. Cell numbers were counted at the end of experiments and normalized the cell concentration as 10^6^ cells/ml culture supernatant. The TGFβ concentration was presented as pg/ml.

### Quantitative reverse-transcription polymerase chain reaction (RT-PCR)

Total RNA was isolated from cultured cells, colonoids, and colon tissues, using an RNA isolation kit (Qiagen, Valencia, CA) and was treated with RNase-free DNase. Reverse transcription was performed using the High-Capacity cDNA Reverse Transcription kit and the 7300 RT-PCR System (Applied Biosystems, Foster City, CA). The data were analyzed using Sequence Detection System V1.4.0 software. Primers, Setd1b (Mm00616971, Hs00324585), Tgfb1 (Mm01178820, Hs00998133), TNF (Mm00443259), and IL6 (Mm00446190), KC (Mm00433859), GAPDH (Mm4352339E, Hu4310884E) were purchased from Applied Biosystems. The relative abundance of GAPDH mRNA was used to normalize levels of the mRNAs of interest. All complementary DNA samples were analyzed in duplicates.

### Immunofluorescence staining

Paraffin-embedded human colonoid and colon tissue sections were deparaffinized followed by antigen retrieval by boiling in citrate acid buffer (#S23045–500, Research products International Corporation) in a pressure cooker at 105°C Sections were blocked using 10% goat serum for 1 h at room temperature. Sections were stained with ZO-1, H3K4me1, and MPO by incubation with a rabbit anti-mouse ZO-1 (#61–7300, Invitrogen Life Technologies) antibody, a rabbit polyclonal anti-H3K4me1 antibody (#5326, Cell Signaling), and anti-MPO (#MPO-121-FITC, FabGennix International Incorporated) overnight at 4°C, followed by a FITC-labeled goat anti-rabbit IgG (#111-165-003, Jackson ImmunoResearch) antibody or Cy3-labeled anti-mouse (#115-165-003, Jackson ImmunoResearch) antibody at room temperature for 2 h. Sections were then mounted using Mounting Medium containing DAPI for nuclear counter-staining. Slides were scanned using the Apiro Versa 200 platform or observed using Leica DM IRB inverted microscope and images were recorded using an Echo/Revolve (CD230RevA) camera.

### Western blot analysis

To harvest protein lysates, cell pellets were solubilized in cell lysis buffer containing 1% Triton X-100 (#T8787, Sigma-Aldrich, St Louis, MO), 50 mmol/L Tris (pH 7.4), 1 mmol/L EDTA, 150 mmol/L NaCl, and a protease and phosphatase inhibitor mixture (#PPC2020, Sigma-Aldrich), to obtain total cellular lysates. The protein contents of lysates were determined by BCA protein assay. The lysates were mixed with Laemmli sample buffer (#1610737, Bio-Rad, CA, USA), and equal amounts of protein were loaded. Proteins in the lysates were resolved by SDS-PAGE and transferred to polyvinylidene difluoride membranes. Western blot analysis was performed using anti-total SMAD2 (5339; Cell Signaling Technology), anti-phospho-SMAD2 (3108; Cell Signaling Technology), anti-H3K4me3 (#9751S, Cell Signaling Technology), anti-H3K4me1 (#5326S, Cell Signaling Technology) and anti-histone 3 (#9715S, Cell Signaling Technology), and anti-β-actin (#A2228, Sigma-Aldrich) antibodies. The band density was measured using the ImageJ (National Institutes of Health, Bethesda, MD) processing program. The relative band density by a specific antibody was calculated by comparing it with the β-actin band from the same sample. The relative band density in the control group was set as 1. The density fold change was obtained by comparing the relative band density in the treatment group with that in the control group.

### Statistical analysis

Statistical significance was determined by One-way Anova analysis of variance for multiple comparisons and the t-test for comparing data from two samples using Prism 9.0 (GraphPad Software, Inc, San Diego, CA). A *P* value less than 0.05 was defined as statistically significant. All data are presented as means ± SD. Results from *in vitro* studies shown in this manuscript represent data from at least three independent experiments. Data from all mice in this study were included in the analysis.

## Results

### The three-dimensional model of p40 and generation of recombinant p40 peptides

The structure of biomolecules, particularly the three-dimensional structure of proteins, can provide crucial information connecting the genomic sequence to its corresponding unique functions. p40 originally purified from LGG culture supernatant is a 412 amino acid secretory protein.^12^ Domain analysis using the NCBI Conserved Domain Database^29^ indicated that the p40 N-terminal region (1–263 residues) is an uncharacterized domain with unknown function and the C-terminal region (303–390 residues) exhibits high homology to cysteine, histidine-dependent amidohydrolase/peptidase (CHAP). Consistent with this, several reports have shown that p40 has hydrolase and muramidase activities.^13,30,31^

**Figure 1.**
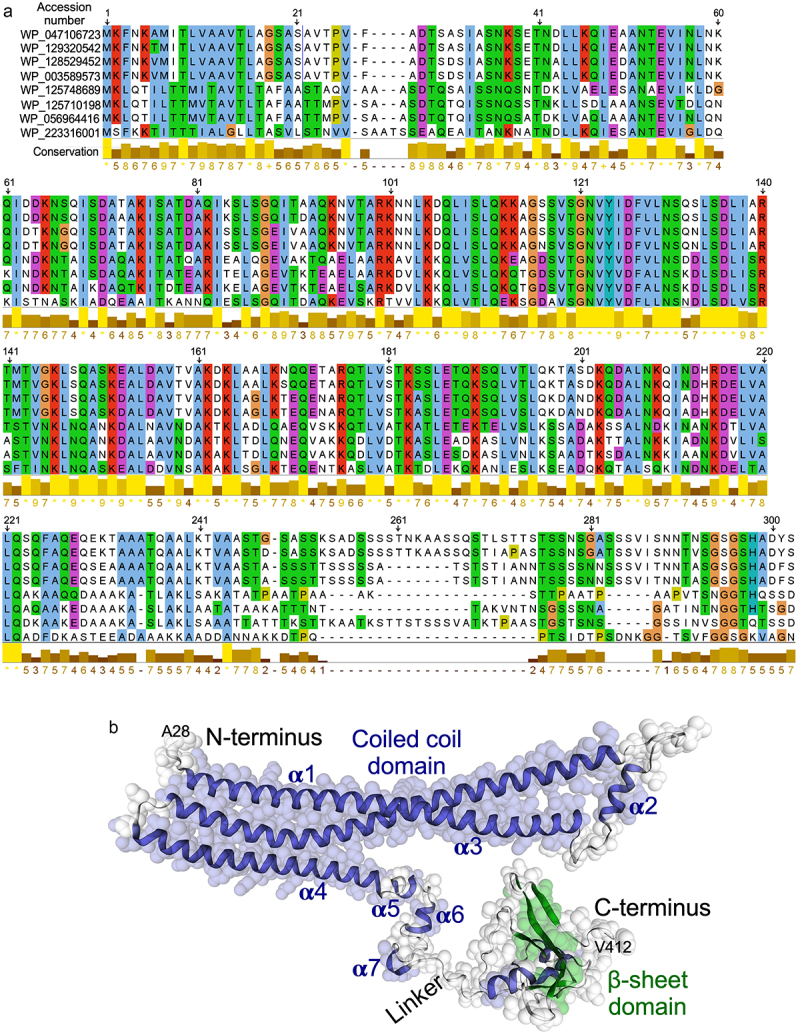
Multiple sequencing alignment and three-dimensional structure of p40. (a) multiple sequencing alignment of p40 N-terminal 1–302 residues was performed using MEGA 11. Structural homology was found among *Lacticaseibacillus* genus. WP_047106723.1: LGG (p40 sequence); WP_129320542.1: lacticaseibacillus chiayiensis, WP_128529452.1: Lacticaseibacillus paracasei; WP_003589573.1: Lacticaseibacillus paracasei; WP_125748689.1: lacticaseibacillus baoqingensis, WP_125710198.1: Lacticaseibacillus porcinae; WP_056964416.1: lacticaseibacillus manihotivorans; WP_223316001.1: Latilactobacillus curvatus. (b) three-dimensional structure of p40 protein predicted by using SWISS-MODEL, alpha-fold, and i-TASSER.

Next, a computational tool, Molecular Evolutionary Genetics Analysis 11 (MEGA 11) was employed for MSA of p40 N-terminal 1–302 residues with unknown function. The MSA results showed that the initial 28 amino acids are the characteristic secretory signal sequence motif, thus were predicted to code for a signal peptide. This signal peptide is highly conserved in the cell wall hydrolase and CHAP domain-containing protein family. The p40 amino acid sequence was found to have a high degree of structural homology among the *Lacticaseibacillus* genus (Figure 1a). The conserved sequences and motif annotation showed that the sequence from 187 to 261 belongs to ATP synthase subunit D (PF01813).

The *in silico* analyses including SWISS-MODEL, Alpha-fold, and i-TASSER were applied to predict the three-dimensional model of p40 from the protein sequence. The overall structure describes the 94.60% Ramachandran-favored area, indicating the robustness of the three-dimensional structure prediction for p40. Consistent with multiple sequencing alignment results, the p40 N-terminal 1–28 residues represent a signal sequence for protein secretion. p40 is composed of three distinct regions: a coiled coil domain in the N-terminus, a β sheet structure in the C-terminus, and a linker region connecting the *N*- and C-terminus (Figure 1b). The N-terminal coiled coil domain contains seven helices, three long helices (α1, α3 and α4) and four short helices (α2, α5, α6 and α7). The linker region is composed of an alanine-rich region. It is anticipated with a high level of confidence that p40 C-terminal residues (Ser302-Gly389), having high homology to the CHAP domain, may fold into a β sheet structure.

This structure encapsulates the key features of the protein, enabling a comprehensive understanding of its structural characteristics and potential functional implications. To ensure accessibility, the predicted three-dimensional structure of p40 was shown in a machine-readable format. Specifically, we have formatted the structure according to the industry-standard Protein Data Bank (PDB) format. This format allows for the accurate representation of atomic coordinates, annotations, and relevant metadata associated with the protein structure (Supplemental data 2).

**Figure 2.**
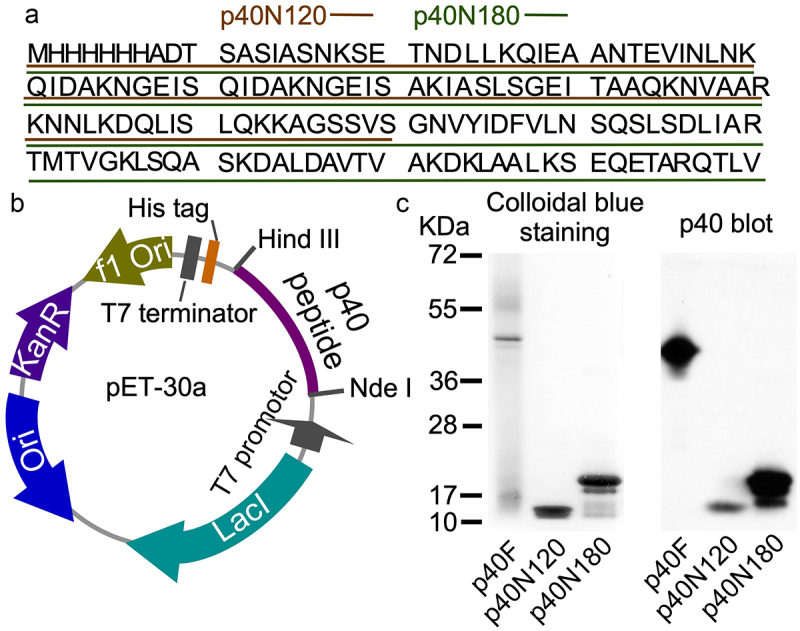
Generation of recombinant p40 peptides. (a) amino acid sequences of p40N120 and p40N180. (b)pET30a DNA vector used to synthesize recombinant p40 peptides. (c) p40F, p40N180 and p40N120 were separated by SDS-PAGE and stained with Colloidal blue staining kit and were analyzed by Western blot analysis using an anti-p40 antibody.

The first three alpha helices are arranged with the first alpha helix (residues Lys46-Gln112) connecting to the second and third alpha helices (residues Thr141-Ile211) through a linker β sheet region (residues Lys113-Ser120 and Ser131-Arg140) (Figure 1b). Functional annotation using the GenomeNet (https://www.genome.jp/tools-bin/search_motif_lib) indicated that the N-terminal region of p40 protein is functionally active. However, this region strongly displays as domain of unknown function (DUF), including DUF881 ranging between 8 and 94 residues and DUF1951 between 100 and 118 residues. Based on these findings, we generated two His-tagged p40 recombinant peptides, p40N120 (28–120 residues) with α1 and α2 and p40N180 (28–180 residues) with α1, α2, and α3 Figure 2(a,b). Full-length p40 (p40F) purified from LGG culture supernatant as previously reported^12^ was used as a control. p40N120 and p40N180 migrated as apparent molecular masses of 12 kDa and 18 kDa in the SDS-PAGE gel, respectively, and were recognized by an anti-p40 antibody (Figure 2c).

### p40N120 is sufficient to stimulate epigenetic program in intestinal epithelial cells by using in vitro assays

**Figure 3.**
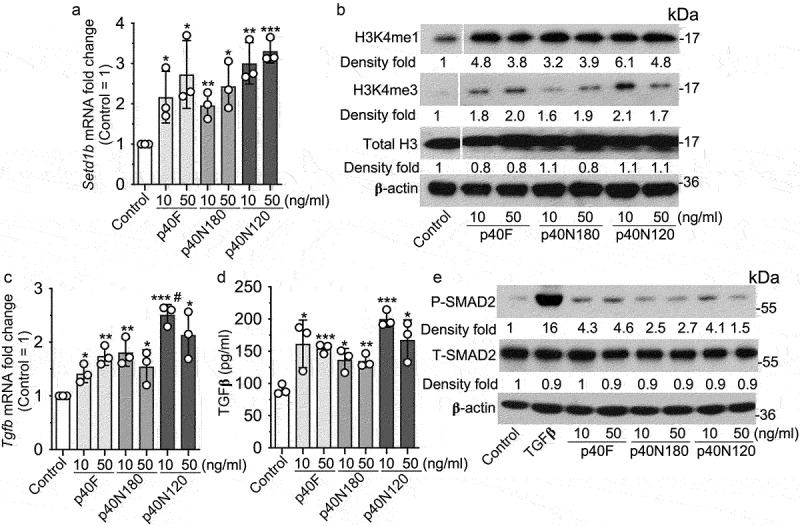
p40N120 upregulates epigenetic program in mouse intestinal epithelial cells *in vitro*. YAMC cells were treated with p40F, p40N180 and p40N120 at 10 or 50 ng/ml for 4 (a, c, d) or 2 (b, d) hours or TGFβ at 10 ng/ml for 15 minutes (e). (a, c) RNA was isolated from cells for RT-PCR analysis of *Setd1b* and *Tgfb1* mRNA levels. *Setd1b* and *Tgfb1* mRNA expression levels in control groups were set as 1. (b, e) total cellular proteins were prepared from YAMC cells for Western blot analysis using indicated antibodies. β-actin blot was used as the protein loading control. The fold changes of band intensities compared to β-actin in the same sample are shown under the blot. (d) YAMC cell culture supernatants were collected for measuring the amount of TGFβ release using ELISA. TGFβ concentration is presented in picograms per milliliter. **p < .001, *p < .01 compared the control group. ^#^*p* < .01 compared to the p40F group. In *A, C*, and *D*, each symbol represents data from one independent experiment. In *B*, lanes were run on the same gel but were noncontiguous, as indicated by the white lines. P-SMAD2: phosphorylated; T-SMAD2: total SMAD2.

We have shown that p40 promotes sustained TGFβ production in intestinal epithelial cells through epigenetic modification, specifically, upregulation of expression of Setd1β, a methyltransferase for assembly and regulation of histone H3 on the lysine 4 mono- and trimethylation (H3K4me1/3).^19^ To study the roles of p40 peptides, we first performed *in vitro* analysis using young adult mouse colonic (YAMC) epithelial cells to determine the effects of p40 peptides on epigenetic modification. Expression of *Setd1b* was significantly upregulated in YAMC cells following p40F, p40N180, and p40N120 treatment in concentration-dependent manner (Figure 3a). Furthermore, the increase in *Setd1b* was corresponded to an increase in H3K4me1 and H3K4me3 in YAMC cells after treatment with p40F, p40N180 and p40N120, as determined by Western blot analysis (Figure 3b). One target of the p40-regulated epigenetic program in intestinal epithelial cells is the *Tgfb1* locus promoting a transcriptionally permissive chromatin state driving gene expression.^19^ p40F, p40N180 and p40N120 significantly stimulated *Tgfb1* gene expression in YAMC cells (Figure 3c) that corresponded to an increase in TGFβ protein in YAMC culture supernatants (Figure 3d) and activated SMAD2, a TGFβ-regulated target (Figure 3e).

**Figure 4.**
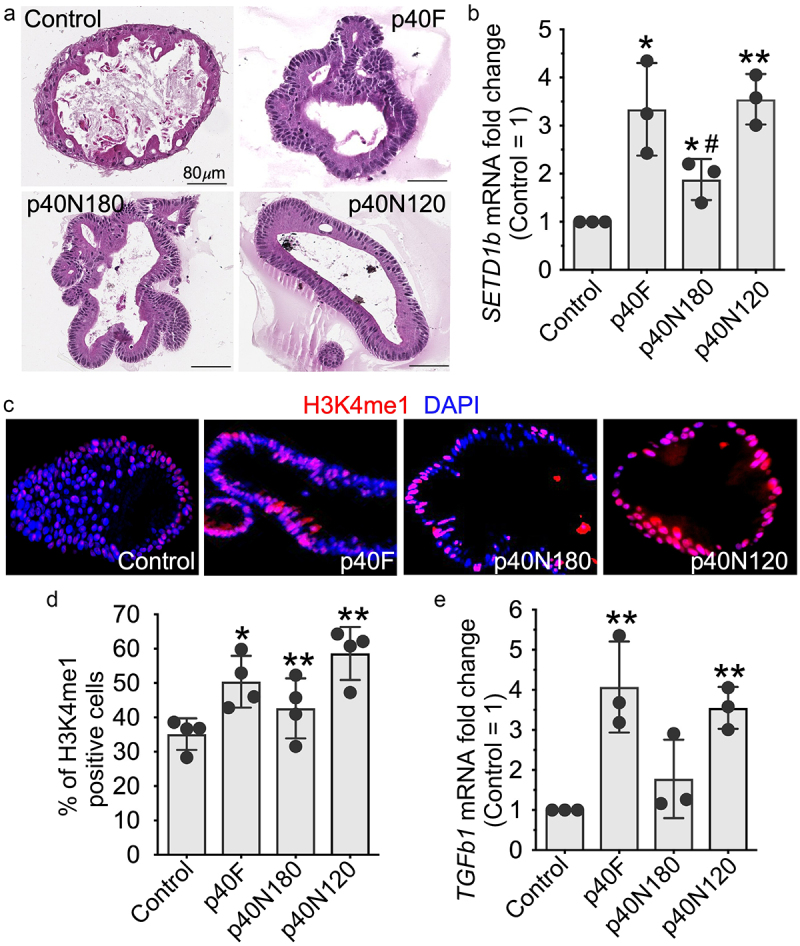
p40N120 stimulated epigenetic responses in human intestinal epithelial cells. Human colonoids were treated with p40F, p40N180 or p40N120 at 100 ng/ml for 48 hours. (a) colonoids were fixed and stained with hematoxylin and eosin. (b, e) RNA was isolated from colonoids for RT-PCR analysis of the levels of *SETD1b* and *TGFb1* mRNA. Expression levels of *SETD1b* and *TGFb1* mRNA in control groups were set as 1. The mRNA expression levels of treatment groups were compared to the control group. (c, d) colonoid sections were stained using anti-H3K4me1 antibody and Cy3-labeled secondary antibody (red). Nuclei were stained with DAPI (blue). % of H3K4me1 positive cells is shown. **p < .001, *p < .01 compared the control group. ^#^*p* < .01 compared to the p40F group. Each symbol represents data from one independent colonoid culture experiment.

Using a relevant translational system, we evaluated the effects of p40 peptides in a human colonoid culture system (Figure 4a). Similar to observations in YAMC cells, p40F, p40N180 and p40N120 significantly up-regulated *SETD1B* (Figure 4b) and increased H3K4me1 positive cells (Figures 4(c,d)) as well as *TGFB1* gene expression (Figure 4e). This finding indicates that the p40N120 peptide is sufficient to stimulate this epigenetic program in intestinal epithelial cells and likely contains the functional domain of p40.

### p40N120 has long-lasting effects on TGFβ production and Treg differentiation in mice

**Figure 5.**
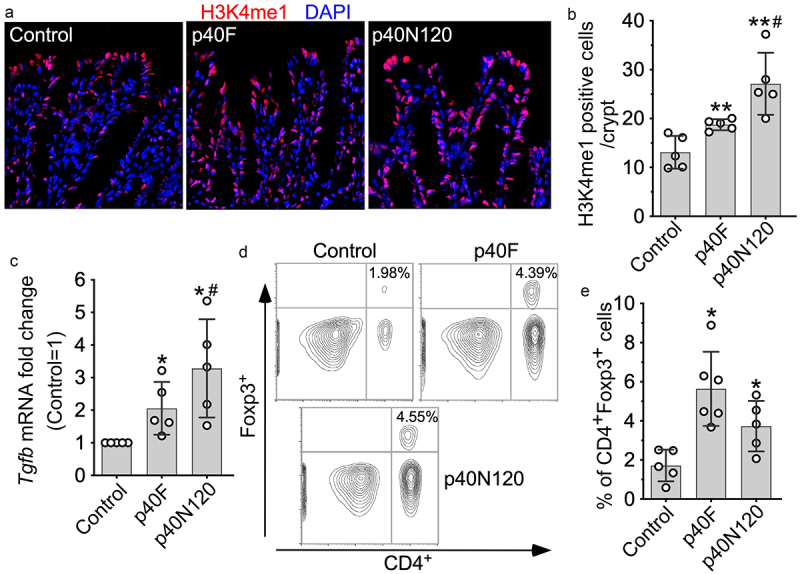
p40N120 supplementation in early life promotes TGFβ production and Treg differentiation in the lamina propria of the colon in adult mice. Foxp3-GFP mice were treated with p40F or p40N120 from postnatal day 2 to day 21. Mice were euthanized at the age of 8 weeks. Paraffin-embedded colon tissues were used to determine H3K4me1 expression by immunohistochemistry using anti-H3K4me1 antibody and Cy3-labeled secondary antibody (red). Nuclei were stained with DAPI (blue). Images were taken using fluorescent microscope at 40X. (b) the number of H3K4me1 positive cells per crypt is shown. (c) RNA was isolated from the colonic tissues for RT-PCR analysis of the *Tgfb1* mRNA expression levels. (d) lymphocytes were isolated from the lamina propria of the colon. Representative counter plot of Foxp3 and CD4 are shown. (e) the percentages of CD4^+^ Foxp3^+^ cells in total lymphocytes were shown. **p < .001, *p < .01 compared the control group. ^#^*p* < .01 compared the p40F group. Each symbol represents data from one mouse (b and c) or from 2–3 mice (e).

We next examined the long-lasting effects of p40N120 on TGFβ production *in vivo*. Forkhead box P (Foxp)3-green fluorescent protein (GFP) pups received p40F and p40N120 daily from postnatal day 2 to 21 and mice were euthanized at 8 weeks. We then quantified H3K4me1 levels in colonic epithelial cells of mice by immunostaining and found a sustained increase in H3K4me1 in adult mice receiving p40F and p40N120 supplementation early in life (Figure 5(a,b)). Remarkably, neonatal p40F and p40N120 supplementation induced long-lasting effects on increasing *Tgfb1* gene expression in the colonic tissues of adult mice (Figure 5c). TGFβ is known to promote the induction of intestinal Treg cells and represents one mechanisms through which the gut microbiota contributes to maintaining intestinal homeostasis.^32^ Therefore, we examined Treg cells induction by flow cytometric analysis of CD4^+^FOXP3^+^ cells in the colonic lamina propria of adult *Foxp3**E*^GFP^ mice. Neonatal p40F and p40N120 supplementation induced sustained increase in Treg cells in the colonic lamina propria (Figure 5(d,e)). As compared to neonatal p40F supplementation, neonatal p40N120 supplementation showed significant higher effects on H3K4me1 (Figure 5b) and *Tgfb1* gene expression (Figure 5c), but not Treg expansion in colonic tissues (*p* > .05, Figure 5(d,e)). This raises the possibility that p40N120 is sufficient to elicit the same or higher level of the long-lasting effect on Treg cell induction in the intestine as compared to p40F.

### Neonatal p40N120 supplementation ameliorates DSS-induced colonic injury/inflammation in adult mice

**Figure 6.**
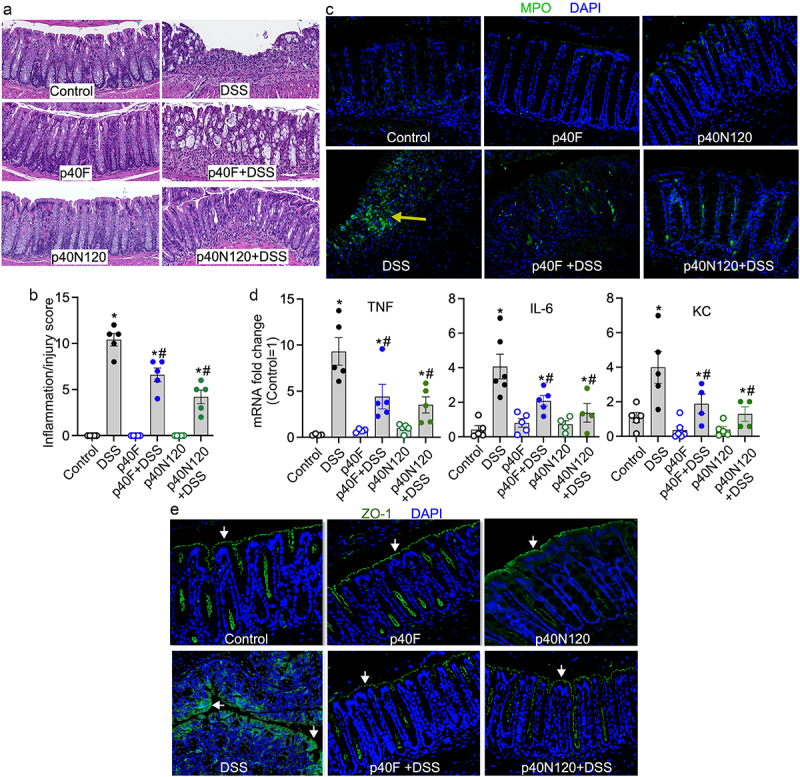
Neonatal p40N120 supplementation prevents colitis in adult mice. Wild-type C57BL/6J mice were treated with p40F or p40N120 from postnatal day 2 to day 21. Colitis was induced by 3% DSS in drinking water for 4 days in mice at the age of 8 weeks. Mice receiving water were used as controls for DSS treatment. (a) colon sections were stained with hematoxylin and eosin for assessment of inflammation. (b) the inflammation/injury scores are shown. (c, e) MPO expression (*yellow arrows*) and ZO-1 localization (*white arrows*) in colonic sections were evaluated by immunohistochemistry using anti-MPO and anti-ZO-1 antibodies, respectively, followed by a FITC-labelled secondary antibody (green). Nuclei were stained with DAPI (blue). (d) RNA was isolated from the colonic tissues for RT-PCR analysis of the indicated proinflammatory cytokine mRNA levels. The average of mRNA expression levels in the control mice was set as 100%, and the mRNA expression level of each mouse was compared to this average. *p < .05 compared to the control mice. ^#^*p <* .05 compared to the DSS control group. Each symbol in *B* and *C* represents data from one mouse. In *D* and *E*, images represent data from 5 mice in each group.

C57BL/6 pups received p40F or p40N120 from postnatal day 2 to 21. At 8 weeks of age, mice were administered 3% DSS in the drinking water for 4 days to induce acute injury/inflammation in the colon. DSS-induced colitis in mice with colon ulceration, crypt damage, and inflammatory cell infiltration (score: 10.40 ± 0.68), which was mitigated by neonatal supplementation with p40F (6.6 ± 0.75, *p* < .05) and p40N120 (4.2 ± 1.0, *p* < .05) (Figure 6(a,b)). Neutrophil-myeloperoxidase (MPO) is an abundant enzyme that catalyzes the production of reactive oxygen species and are biomarkers of oxidative damage that is increased in the intestinal mucosa of patients with ulcerative colitis. Colonic MPO was assessed by immunostaining. Adult mice that received p40F and p40N120 supplementation in early life exhibited fewer MPO positive cells compared to mice given DSS without supplementation (Figure 6c). DSS stimulated expression of proinflammatory cytokines including tumor necrosis factor (TNF), interleukin-6 (IL-6), and keratinocyte chemoattractant (KC) were significantly reduced in the colon of mice treated as neonates with p40F and p40N120 supplementation (Figure 6d). DSS-induced colitis was characterized by disruption of the intestinal epithelial integrity visualized by the distribution of the tight junctional protein zonula occludens-1 (ZO-1) via immunostaining. Neonatal p40F and p40N120 supplementation prevented DSS-induced redistribution of ZO-1 from the apical tight junction complex to the cytoplasm of colon epithelial cells in adult mice (Figure 6e). Collectively, these data suggest that p40 peptide containing residues 28–120 harbor the functional domain essential for sustained TGFβ production and Treg cell induction that promotes long-lasting effects toward preventing experimental colitis in mice.

## Discussion

Production of functional factors by probiotics is an emerging strategy to exert beneficial effects on the host.^8,11^ To advance the mechanistic knowledge and enable broad application of these functional factors, it is crucial to elucidate the structure-functional relationship of these functional factors. However, experimental strategies to obtain three-dimensional structures of bacterial-derived proteins are challenging. Newer powerful computational tools such as SWISS-MODEL, Alpha-fold, and i-TASSER used in this study make it possible to derive probable structural information with high accuracy from the genomic sequences. Remarkably, the three-dimensional model of p40 generated by this approach shows high similarity to the crystal structure of a *Streptococcus pneumoniae*-derived protein, PscB,^23^ which has high sequence homology to p40. PscB has been reported to be composed of three distinct regions, a coiled coil (41–266 residues), a linker region (267–278 residues) and a CHAP domain (279–392 residues).^23^ The coiled coil domain of PscB is arranged in five helices, three long helices (α1, α3 and α4) composed of 66, 73 and 41 residues, respectively, and two short helices (α2 and α5).^23^ Further, PcsB adopts a dimeric structure.^23^ Our structural model also predicted that p40 is present as a tetramer protein. Therefore, the PscB model from the crystal structural studies supports the p40 structural model identified by this work.

The structure-based findings from this work provide insights into understanding the mechanisms of the action of p40. For example, the coiled-coil structure is known to facilitate dimerization of DNA-binding transcriptional factors.^33^ Our LC-MS-MS proteomic analysis showed that p40 isolated from LGG culture supernatant was co-immunoprecipitated with Max, Mga, and myosin 9 and 10 from YAMC cells. Max and Mga are transcriptional factors and the dimerization of Mga and Max is required for their transcriptional activities. Then, we generated His-tagged p40F. We further verified that Max and Mga in YAMC cells were co-precipitated with p40N and p40N120 by Western blot analysis (Supplemental Figure S1A). Importantly p40F and p40N120 promoted the Mga:Max dimerization (Supplemental Figure S1B). This finding strongly suggests that p40 interacts with the Mga:Max complex to stimulate gene expression. p40 may directly interact with both Mga and Max, or with Max or Mga. The endpoint of this interaction is to enhance the Mga:Max dimerization. The helix structure of p40 may be responsible for the interaction between p40 and the Mga:Max complex, particularly, α1 and α2 helix in p40N120. Our future works will be focused on elucidating the role of p40-regulated Mga:Max dimerization in *Setd1*β gene expression.

The structure-functional studies in this work first reveal the functional peptide of a probiotic-derived factor for upregulating TGFβ production and stimulating TGFβ signaling in intestinal epithelial cells. These findings open a new avenue for probiotic application because activation of TGFβ signaling plays multiple roles in health, including protection of the intestinal epithelium against insults and suppressing inflammation by the induction of Treg cells.^34–37^ It has been reported that *Limosilactobacillus mucosae* showed greater efficacy than *Lactobacillus amylovorus* in alleviating DSS-induced colonic inflammation, which was coordinated with TGFβ production and serotonin receptors in the colon. This finding suggests that regulation of TGFβ may be one of the most important mechanisms underlying the probiotic effects of lactobacilli in gut inflammation.^38^ TGFβ signaling has been shown to be highly related to IBD. The locus encoding SMAD3, an effector of TGFβ signaling, is associated with IBD susceptibility.^39^ Blocking TGFβ signaling by a negative regulator, SMAD7, has been shown to increase the chronic production of proinflammatory cytokines and increase in SMAD7 has been found in Crohn’s disease patients.^40^ Further, a randomized, double-blind clinical trial has revealed that treatment with an antisense oligodeoxynucleotide against the Smad7 mRNA transcript leads to endoscopic improvement and remission in patients with Crohn’s disease.^41^ Therefore, the finding that p40N120 stimulates TGFβ production supports the significant clinical potential of p40N120 as a potential IBD therapeutic. Further, application of probiotic-derived factors could bypass the limitations of clinical application of probiotics, such as uncertain bioavailability and biopharmacology of probiotics in the human gastrointestinal tract. The functional peptides of probiotic functional factors may exhibit more priorities, such as efficacy of absorption by host cells and stability in the gastrointestinal tract.

There are several limitations of this study. p40N120 (28–120 residues) containing the first two coiled coils exerts the similar sustained anti-inflammatory effect as p40F. We cannot exclude the possibility that the functional domain may be a shorter peptide within the 28–120 residues of p40. Future studies will utilize a reductionist approach to further define the minimal regions such as the first or the second coiled coil containing peptide for biological functions. Currently, there are no animal models that precisely represent the pathology of patients with IBD. The colitis model used in the current study was limited to the well-established DSS model with colonic injury and acute colitis. Additional research will elucidate the role of p40F and functional peptides in various inflammatory models, such as 2,4,6-Trinitrobenzenesulfonic acid (TNBS)-induced Th1-driven colitis and oxazolone-induced Th2 driven colitis. In addition, other delivery systems that could increase the efficacy of p40 and peptides delivered to the colon and protect their bioactivity are worthy to be investigated. Although we found the p40F and p40N120 stimulated epigenetic responses in normal human intestinal epithelial cells, the insights into their effects on human colonoids from IBD patients remain limited.

In summary, our findings demonstrate that p40N120 containing the coiled coil structure serves as a functional peptide to perform the same function as full length of p40 for upregulating expression of a methyltransferase *Setd1b* for sustained TGFβ production and Treg differentiation in the colon, which likely contributes to a long-lasting protective response against intestinal injury and inflammation. Therefore, the p40 functional peptide has the potential for nutritional and clinical application for maintaining intestinal health.

## Supplementary Material

Supplemental Material

## Data Availability

The authors confirm that the data supporting the findings of this study are available within the article.
