# Supplementary material for: Identification of a functional peptide of a probiotic bacterium-derived protein for the sustained effect on preventing colitis
Source: Gut Microbes. 2023 Oct 10;15(2):2264456. doi: 10.1080/19490976.2023.2264456 (PMC10566403; doi:10.1080/19490976.2023.2264456)
Supplement: Supplemental Material [file KGMI_A_2264456_SM5546.docx]

Supplemental Figure 1

A Blot kDa B


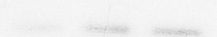

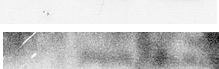

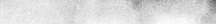

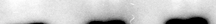

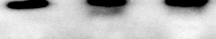


Blot kDa

IP:

Max Mga

-28

-460

-268


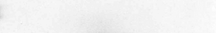

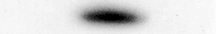

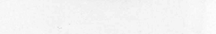

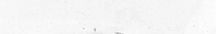

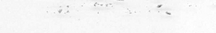

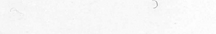

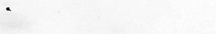

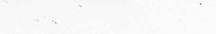

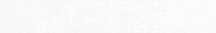

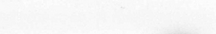

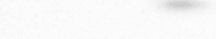


-55

-36

-17

-10

p40F

IP:

Max

Max Mga


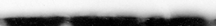

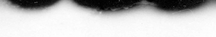

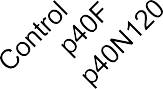


-28


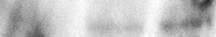

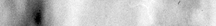


-460

-268

His

p40

Input 𝛃-actin -36

Input

𝛃-actin

p40N120

-36


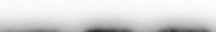

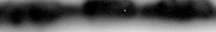

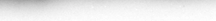

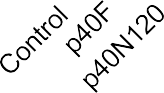


**Supplemental Figure 1. p40 interacts with Max and Mga and increases Mga-Max dimerization in YAMC cells.** YAMC cells were treated with His-tagged p40F and His- tagged p40N120 at 100 ng/ml for 1 hour. (A) Cells were solubilized in 50 mM sodium phosphate buffer, pH 8.0, containing 500 mM sodium chloride 0.5% Triton X-100, 10% glycerol and 10 mM imidazole. Then, soluble proteins (2 mg) were used for immunoprecipitation using Dynabeads His-Tag Isolation & Pulldown (10103D, Invitrogen), according to the manufacturer’s instruction. Proteins binding to dynabeads were solubilized using Laemmli sample buffer for Western blot analysis with anti-p40, anti-Max (4739, Cell Signaling Technology) and anti-Mga (19854, Abcam, Inc.) antibodies. (B) Cells were solubilized in cell lysis buffer. soluble proteins (2 mg) were used for immunoprecipitation using anti-Max antibody and Protein A/G-Plus Agarose (sc2003, Santa Cruz Biotechnology, Inc.). Proteins conjugated to beads were solubilized with Laemmli sample buffer for Western blot analysis using anti-Max and anti-Mga antibodies. Data are from at least 3 independent experiments.
